# Complement activation and blockade in massive post-partum haemorrhage, thrombotic microangiopathy and acute kidney injury: a case report

**DOI:** 10.1186/s12882-021-02456-1

**Published:** 2021-07-06

**Authors:** G. Guzzo, S. Kissling, G. Pantaleo, M. Pascual, S. Sadallah, D. Teta

**Affiliations:** 1 Service of Nephrology, Valais Hospital, Avenue du Grand-Champsec 86, 1950 Sion, Switzerland; 2 Organ Transplant Center, Lausanne University Hospital, University of Lausanne, Rue du Bugnon 46, 1011 Lausanne, Switzerland; 3 Service of Immunology and Allergy, Lausanne University Hospital, University of Lausanne, Lausanne, Switzerland; 4 Service of Nephrology and Hypertension, Lausanne University Hospital, University of Lausanne, Lausanne, Switzerland

**Keywords:** Acute kidney injury (AKI), Bb factor, Complement activation, Complement blockade, Eculizumab, Post-partum haemorrhage (PPH), sC5b-9, Thrombotic microangiopathy (TMA), Case report

## Abstract

**Background:**

Thrombotic microangiopathy (TMA)-mediated acute kidney injury (AKI) following massive haemorrhage is a rare but severe complication of the post-partum period. It is associated with a poor renal prognosis and a high risk of end-stage kidney disease. Complement activation may occur in this picture. However, whether complement activation, and thus complement blockade, may be critically relevant in this setting is unknown.

**Case presentation:**

A 50 year-old woman presented with massive delayed post-partum haemorrhage (PPH). Despite bleeding control and normalization of coagulation parameters, she rapidly developed AKI stage 3 associated with dysmorphic microhematuria and proteinuria up to 2 g/day with the need of renal replacement therapy. Blood tests showed signs of TMA associated with markedly increased sC5b-9 and factor Bb plasma levels, respectively markers of terminal and alternative complement pathway over-activation. This clinical picture prompted us to initiate anti-C5 therapy. sC5b-9 normalized within 12 h after the first dose of eculizumab, factor Bb and C3 after seven days, platelet count after nine days and haptoglobin after 3 weeks. The clinical picture improved rapidly with blood pressure control within 48 h. Diuresis resumed after three days, kidney function rapidly improved and haemodialysis could be discontinued after the sixth and last dose. Serum creatinine returned to normal two years after presentation.

**Conclusions:**

We suggest that massive PPH induced major activation of complement pathways, which ultimately lead to TMA-induced AKI. Various causes, such as oocyte-donation, the potential retention of placental material and the use of tranexamic acid may have contributed to complement activation due to PPH. The prompt administration of anti-C5 therapy may have rapidly restored kidney microcirculation patency, thus reversing signs of TMA and AKI. We propose that complement activation may represent a major pathophysiological player of this complication and may provide a novel therapeutic avenue to improve renal prognosis in TMA-induced AKI following massive PPH.

## Background

Thrombotic microangiopathy (TMA) is a rare but severe complication of pregnancy and post-partum period [1]. Different noxious stimuli may trigger thrombi formation into small vessels leading to thrombocytopenia, anemia due to erythrocyte fragmentation and ischemic tissue damage [2]. Acute kidney injury (AKI) is a frequent consequence of TMA and is associated with a poor long-term renal prognosis [3].

Massive post-partum haemorrhage (PPH) may be a cause of TMA-mediated AKI, mimicking post-partum pregnancy-associated atypical haemolytic uremic syndrome (p-aHUS). However, severe AKI occurring after massive PPH is multifactorial and may be precipitated by other factors including anti-fibrinolytic drugs through impairment of the patency of the kidney microcirculation [4]. Complement activation during massive PPH and TMA is an interesting finding, but its clinical significance remains unclear. We report the case of a massive PPH with dialysis-dependent AKI, TMA, and complement activation successfully treated with anti-C5 therapy.

## Case presentation

**Fig. 1:**
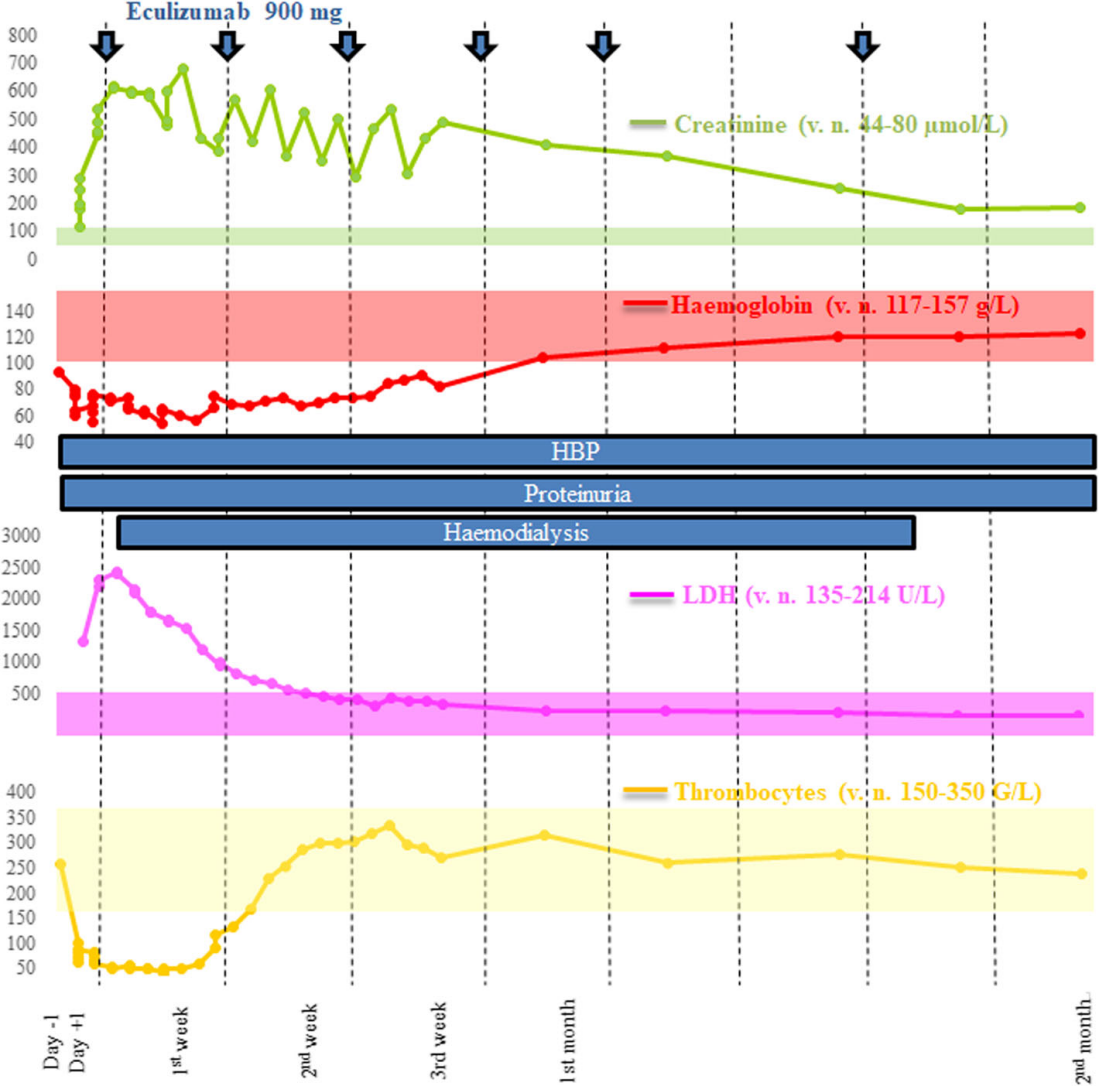
Laboratory and clinical evolution from admission to the end of eculizumab administration. Normal values of creatinine, hemoglobin, LDH and thrombocytes are specified in the figure but also represented by transparent rectangles with the same colors of lines. Eculizumab administration are represented with thick blue arrows. HBP = high blood pressure controlled by therapy

A 50 year-old woman was admitted in our hospital for abdominal pain and massive vaginal haemorrhage. She was at day 9 post-partum, following a non-complicated elective caesarean section after unsuccessful birth induction. This event occurred after a 41 weeks normal gestation resulting from oocyte donation for infertility due to physiological ovarian aging. At admission, blood pressure was maintained at 129/91 mmHg despite massive bleeding. Haemoglobin was 93 g/L with a normal thrombocyte count. She rapidly underwent two uterine curettages followed by embolization of uterine arteries. Treatment also included amines, oxytocine, sulprostone, 2 g of tranexamic acid (TXA), 5 units of packed red blood cells (RBC), 1 unit of platelets, 2 units of frozen plasma and 2 g of fibrinogen for consumption coagulopathy. At Intensive Care Unit admission, serum creatinine was increased to 116 μmol/L and platelet count was decreased to 102 G/L. Due to persistent uncontrolled bleeding, she underwent total hysterectomy, with the administration of 7 additional RBCs and 500 mcg of TXA. Total blood loss was estimated to 2700 mL. Anatomopathological analysis of surgical tissues excluded signs of endometritis. In the recovery room, she developed symptomatic high blood pressure associated with blurred vision, dizziness, headache and hyperreflexia necessitating intravenous labetalol. Despite bleeding control and coagulation markers’ normalization, haemoglobin, platelet count and kidney function continued to worsen, subsequently leading to AKI stage 3 associated with dysmorphic microhematuria and proteinuria up to 2 g/day with the need of replacement therapy. Blood tests showed numerous schizocytes (49‰) with undetectable haptoglobin (< 0.1 g/L) and massive lactate dehydrogenase (LDH 1340 UI/L) levels, clearly defining signs of TMA. Disseminated intravascular coagulation was reasonably ruled out due to a normal fibrinogen level. ADAMTS-13 activity was normal at 38%, although the essay was performed just after the administration of the 2 initial fresh frozen plasma packs. C3 was reduced to 0.63 g/L with markedly increased serum sC5b-9 and Bb factors, respectively to 637 ng/ml (normal range 127–303 ng/mL) and 5.95 μg/mL (normal range <  1.65 μg/mL). Moreover, a serum C3 splitting activity was found giving rise to C3c. Factor H (FH) was normal and Factor I (FI) was slightly but not significantly reduced to 36.2 μg/mL (normal range 38–58 μg/mL), probably due to consumption. No anti-FH antibody was detected (Table 1). The marked activation of complement alternative and terminal pathways prompted us to start anti-C5 therapy, i.e. intravenous eculizumab 900 mg, once weekly, with the required antibiotic prophylaxis and meningococcal vaccination. Interestingly, sC5b-9 normalized within 12 h after the first dose of eculizumab, factor Bb and C3 after seven days, platelet count after nine days and haptoglobin after 3 weeks. The clinical picture improved rapidly with blood pressure control after 48 h and diuresis resumed after three days. Weekly eculizumab was continued aiming a CH50 below 10%. The patient received a total of six doses of eculizumab over two months, until haemodialysis could be discontinued (Fig. 1). Using Next-Generation Sequencing, no complement genetic abnormality in favour of p-aHUS was detected. Two years after admission, the patient was doing well. Serum creatinine was 82 μmol/l, corresponding to an estimated GFR of 71 ml/min/1.73 m^2^ (CKD-EPI Cystatine C), without proteinuria nor haematuria.

**Table 1:** Functional and genetic complement analysis at admission and during follow-up

	Day 0	Day+ 1	Day+ 5	Day+ 8	2nd week	After 9 months
**C3 (normal value 0.76–1.46 g/L)**	0.62 (↓)	–	–	1.04	1.08	–
**C4 (n.v. 0.1–0.4 g/L)**	0.07 (↓)	–	–	0.16	0.18	–
**CH50 (n.v. 70–140%)**	61 (↓)	–	1 (↓)	13 (↓)	2 (↓)	74
**MBL (n.v. > 49%)**	0 (↓)	–	5(↓)	3 (↓)	0 (↓)	–
**AP50 (n.v. > 71%)**	44 (↓)	–	2(↓)	6 (↓)	1 (↓)	76
**C5b-9 (n.v. 127–303 ng/mL)**	637 (↑)	296	245	225.6	–	71 (↓)
**Bb factor (n.v. < 1.65 μg/mL)**	5.95 (↑)	–	2.43 (↑)	1.45	0.98	0.78
**H factor (n.v. 400–800 μg/mL)**	723	–	700	–	803	–
**I factor (n.v. 38–58 μg/mL)**	36.2	–	48.5	–	45.8	–
**B factor (n.v. 213–622 μg/mL)**	308.4	–	–	–	–	–
**Anti-factor H (n.v. < 30 AU/mL)**	< 3.9	–	–	–	–	–
**C3 conversion**	present	–	–	–	–	absent

No genetic abnormality was found in these complement and coagulation genes: ADAMTS13, C3, MCP, CFB, CFD, CFH, CFHR1, CFHR2, CFHR3, CFHR4, CFHR5, CFI, CFP, HOXA2, MMACHC, THBD

## Discussion and Conclusions

We report the case of a woman with massive PPH, TMA-induced AKI and complement major activation, which was not due to a deficit of main regulators of the complement alternative pathway, nor to an anti-Factor H antibody. Genetic screening did not reveal complement variants known to be associated with aHUS. A limitation of our report is the absence of kidney biopsy testifying renal cortical necrosis. This could not be performed due to the patient’s critical conditions. However, in the context of a clear diagnosis of TMA, associated with markedly increased Bb and sC5b-9 factor plasma levels, demonstrating alternative and terminal complement pathway activation, kidney biopsy was not considered as essential for the patient’s clinical management.

**Fig. 2:**
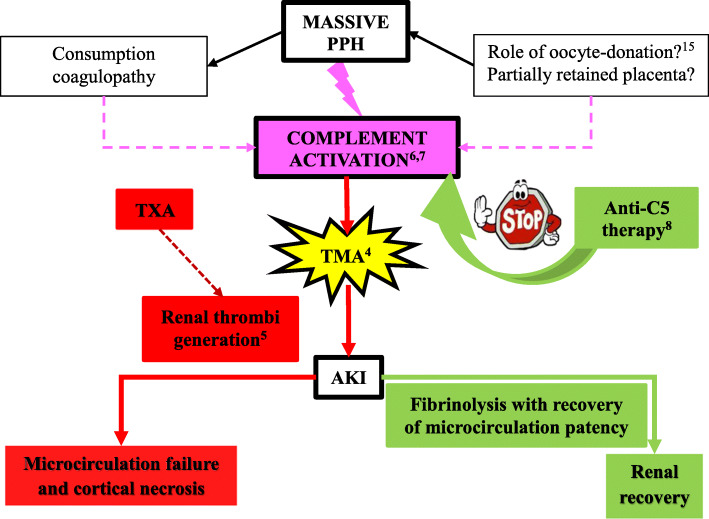
Proposed physio-pathological mechanisms for complement activation in TMA induced-AKI following PPH, with subsequent application of anti-C5 therapy. Blue dotted arrows for potential mechanisms or relations

A recent paper published by Luc Frimat and Alexandre Hertig analysed retrospectively 105 cases of post-partum AKI, admitted in 9 different French intensive care units from 2011 to 2015 [5]. In 14 cases, AKI was attributed to TMA, and in 1 of these 14 patients, TMA was associated with PPH. AKI was related to PPH in 33 women; however, platelets counts rapidly improved at day 3 among these patients, unlike in TMA. Figure 1 clearly shows that our patient had persistently low platelet count after AKI onset, in line with the diagnosis of TMA. Our observation suggests that massive hemorrhage could be an alternative trigger of complement activation in cases of TMA not mediated by genetic or acquired predisposition to aHUS (Fig. 2).

Complement and coagulation cascades mutually belong to the first line of defence against noxious stimuli and descend from a common ancestor. Different animal experimental models showed that haemorrhagic shock can activate complement [6, 7]. Furthermore, complement depletion with anti-C5 therapy or cobra venom factor improved post-resuscitation parameters in experimental model of haemorrhagic shock [6, 8]. In a prospective cohort of 208 adult trauma patients, complement activation and amplification by the alternative pathway, demonstrated by C5b-9 and Bb factor increased levels, were correlated with injury severity, tissue hypoperfusion and worse clinical outcomes, including acute kidney injury [9]. The precise molecular pathway of the cross talk between both cascades remains to be elucidated [10, 11]. Thrombin, factors IXa, Xa, XIa, and plasmin, appear to directly activate complement subunits C3 and C5, and independently of each other [12, 13]. Kallikrein, similarly to factor D, cleaves C3bB generating the alternative pathway C3-convertase (C3bBb) [14].

Various causes may have contributed to complement activation because of PPH in this case. Oocyte-donation has been associated with a higher incidence of PPH but its direct role in complement activation has not been investigated [15]. Retention of placental material may have contributed as a cause of delayed PPH, since complete uterine revision was made difficult due to the abundance of material found. A retrospective analysis of 18 PPH patients complicated by AKI proposed that TXA use was associated with renal cortical necrosis. We propose that TXA which is known to inhibit plasmin and the enzymatic breakdown of fibrinogen and fibrin may have delayed the clearance of vascular microthrombi, and thus TMA resolution [16, 17]. Compared to Frimat’s case series, in which only two patients showed a significant improvement of kidney function at six months and eight remained dialysis-dependent, our patient showed a dramatically better kidney outcome. We suggest that administration of anti-C5 therapy may have prompted the recovery of microcirculation patency and subsequent AKI resolution. In addition, the rapid normalization of sC5b-9 and Bb factor levels, ahead of clinical improvement, appears in line with our hypothesis.

In conclusion, we suggest to explore complement pathways systematically in patients with PPH and TMA-induced AKI. In the case of sC5b-9 and Bb factor elevations, the administration of anti-C5 therapy may prompt rapid normalization of sC5b-9 and thus the recovery of microcirculation patency and subsequent AKI.

## Data Availability

The authors declare that all main data supporting the findings of this study are available within the article.

## Abbreviations

AKI: Acute kidney injury
ESKD: End-stage kidney disease
FH: Factor H
FI: Factor I
LDH: Lactate dehydrogenase
p-aHUS: Pregnancy-associated atypical haemolytic uremic syndrome
PPH: Post-partum haemorrhage
RBC: Red blood cells
TMA: Thrombotic microangiopathy
TXA: Tranexamic acid
